# In-feed bacitracin methylene disalicylate modulates the turkey microbiota and metabolome in a dose-dependent manner

**DOI:** 10.1038/s41598-019-44338-5

**Published:** 2019-06-03

**Authors:** Timothy A. Johnson, Matthew J. Sylte, Torey Looft

**Affiliations:** 1 National Animal Disease Center, Agricultural Research Service, United States Department of Agriculture, Ames, IA 50010 USA; 2 Present Address: Department of Animal Sciences, Purdue University, 270S Russell St., West Lafayette, IN 47907 USA

**Keywords:** Metagenomics, Microbiome

## Abstract

Beginning in 2017, the subtherapeutic use of most antibiotic compounds for growth promotion in food producing animals in the US was prohibited, highlighting the need to discover alternative growth promotants. Identifying the mechanism of action of growth promoting antibiotics may aid in the discovery of antibiotic alternatives. We describe the effects of feeding a subtherapeutic (50 g/ton of feed) and therapeutic (200 g/ton) concentration of bacitracin methylene disalicylate (BMD) to commercial turkeys for 14 weeks, and its effect on turkey intestinal microbial communities and cecal metabolomes. Both BMD concentrations had an immediate and lasting impact on the microbiota structure, and reduced bacterial richness through the end of the study (12 weeks). Metabolomic analysis identified 712 biochemicals, and 69% of metabolites were differentially present in BMD treated turkeys for at least one time point (q < 0.1). Amino acids, carbohydrates, nucleotides, peptides, and lipids were decreased in the turkey ceca early after BMD administration. Long-term metabolome alterations continued even after withdrawal of BMD. The microbial composition, determined by 16S rRNA gene sequencing, was predictive of the metabolome, indicating a connection between the microbiome and metabolome. In-feed BMD may cause bacterial metabolic shifts, leading to beneficial traits that can be targeted to improve animal health and production.

## Introduction

Antibiotics have been used in poultry production for ~70 years. However, in 2017 many antimicrobial compounds previously used in the U.S. in food producing animals were withdrawn for growth promoting applications. This change in governmental regulations has left animal producers searching for antibiotic alternatives to aid in animal health and production, even though the mechanism by which antibiotics improve feed efficiency is still not fully understood^1^. The performance benefits are likely due to reductions and/or shifts in the gut microbiota that may lead to metabolic changes in the gut environment, potentially benefiting host health, thereby improving feed efficiency (ratio of weight gained and feed consumed)^1,2^. Determining the modulated functions within the microbiota of antibiotic altered microbiomes will likely provide insights into the mechanism of action of growth promoting antibiotics and may elucidate alternative approaches to promote animal growth while not providing a selective pressure for antibiotic resistance.

Members of the microbiota produce metabolites that can promote intestinal health^3^ and these beneficial functions may improve animal production. Harnessing these functions may provide alternatives to antibiotics to promote health and growth. Short chain fatty acids (SCFA) and other microbial fermentation end products, induce broad effects such as mucus secretion, improved tight junctions of mucosal epithelium, regulation of T cell function, and even serve as an energy source for colonocytes^3,4^. Tryptophan metabolites, also produced by the microbiota, are important for maintenance of gut immune homeostasis and inflammation control^5^. Non-digestible prebiotics and bacteria with appropriate metabolic genes are required to produce the functions of interests^6^. The microbiota can exert a significant impact on the nutrient utilization^7^ and immunological status and health of the host^8^.

Many studies have evaluated antibiotic effects on the microbiota membership in humans and animals^9–11^, and in some cases explore functional gene diversity (metagenome). While these studies have led to important observations in the microbiota community structure, fewer studies address the functional effects of antibiotics on the microbiota. Metatranscriptomics^12^ and metabolomics^13^ studies explore the gene expression levels and metabolite concentrations, respectively, in the intestinal environment the functional activities of the microbiota. Bacterial biochemicals and metabolites interact with the host, so understanding metabolomic shifts in the microbiome may highlight key compounds associated with improved feed efficiency in animal systems.

Studies exploring relationships between the metabolome of the intestinal tract and the microbiota have identified broad effects. Germ-free and conventional mice have dramatically different blood biochemical profiles, suggesting that metabolomic impacts of the microbiota are not limited to the gut environment^14^. It has been proposed that antibiotics alter the metabolic activity of the microbiota, as detected by increases in metabolites that are normally degraded by bacteria in the mouse gut (i. e. primary bile acids and sugar alcohols)^15^. Fewer studies have explored the metabolome in poultry production systems, though, a recent study profiled the ileal metabolome of 3-week old chickens fed subtherapeutic virginiamycin or bacitracin methylene disalicylate (BMD)^13^. Increases in amino acid and fatty acid metabolites were detected with both antibiotics in the ileum of chickens, but association with the members of the microbiota were not explored. Additional research is needed to evaluate associations between metabolite production and the microbiota in the ceca, in an effort to elucidate the impact of growth promoting antibiotics impact microbiota populations and bacterial-derived metabolites and potential insights on the growth promoting effect of antibiotics.

**Figure 1:**
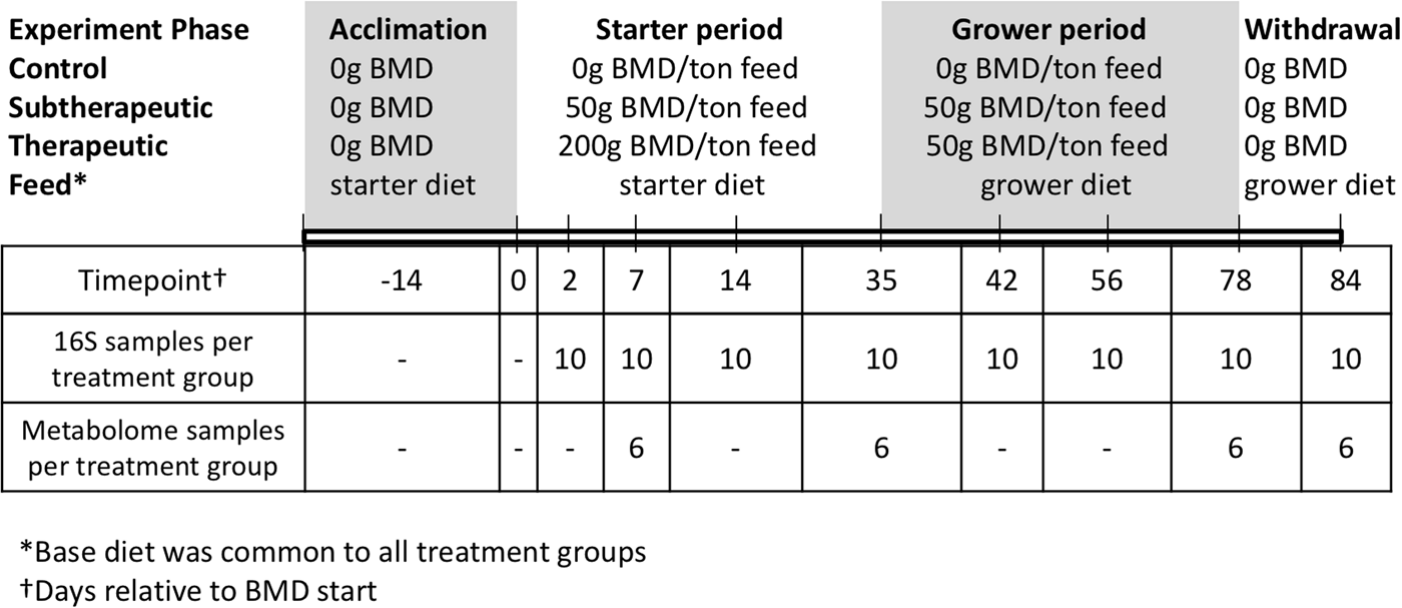
Timeline of animal treatment throughout BMD feed study. Days (relative to start of BMD treatment) when animal feed was changed and when samples were collected. A dash (“−”) indicates that no samples were collected. Diet alterations occurred on days 35 and 78 after samples were collected on those days. BMD, bacitracin methylene disalicylate.

In this study, we describe the effects of therapeutic and subtherapeutic BMD, an in-feed antibiotic additive, on turkey intestinal microbial communities and cecal metabolomes over 14 weeks (Fig. 1). Both concentrations of BMD had immediate and lasting impacts on the microbiota structure, reducing species richness in the BMD-treated poults through the end of the study. Metabolomic analysis identified 712 compounds, including decreased amino acid, protein and lipid content early after BMD administration, as well as lasting alterations of tryptophan metabolites even after withdraw of BMD. The microbial composition was predictive of the metabolome indicating that these temporal effects may be due to an early antibiotic disturbance followed by long-term shift in microbial composition resulting in a shift in bacterial function. Connecting the microbiome structure and metabolomic response during antibiotic disturbance may improve microbiota modulation strategies that can be targeted to improve animal health and production.

## Results and Discussion

In this study, we evaluated the effects of BMD on the bacterial community in the turkey jejunum, ileum, and ceca, as well as the cecal metabolome in turkeys from shortly after hatch until nearly market age. BMD is commonly used for growth promotion, feed efficiency, and disease prevention and treatment both in poultry and swine. Subtherapeutic use of BMD is permitted under the FDA veterinary feed directive^16^, because bacitracin is determined to not be essential for human health. The mechanism of action of how BMD improves animal feed efficiency is unknown (as is the case for all antibiotic growth promotors). The goal of this study was to understand the effects of BMD on the turkey intestinal microbiota and the intestinal metabolomic composition to potentially identify important microbiome functions associated with animal growth promotion (an application of BMD). We observed distinct jejunal, ileal and cecal bacterial communities, shifts in bacterial genera due to BMD treatment, changes in metabolome composition, and correlations between the metabolome and the bacterial community composition.

### Gut location, age, and in-feed BMD treatment all impact the turkey microbiota

**Figure 2:**
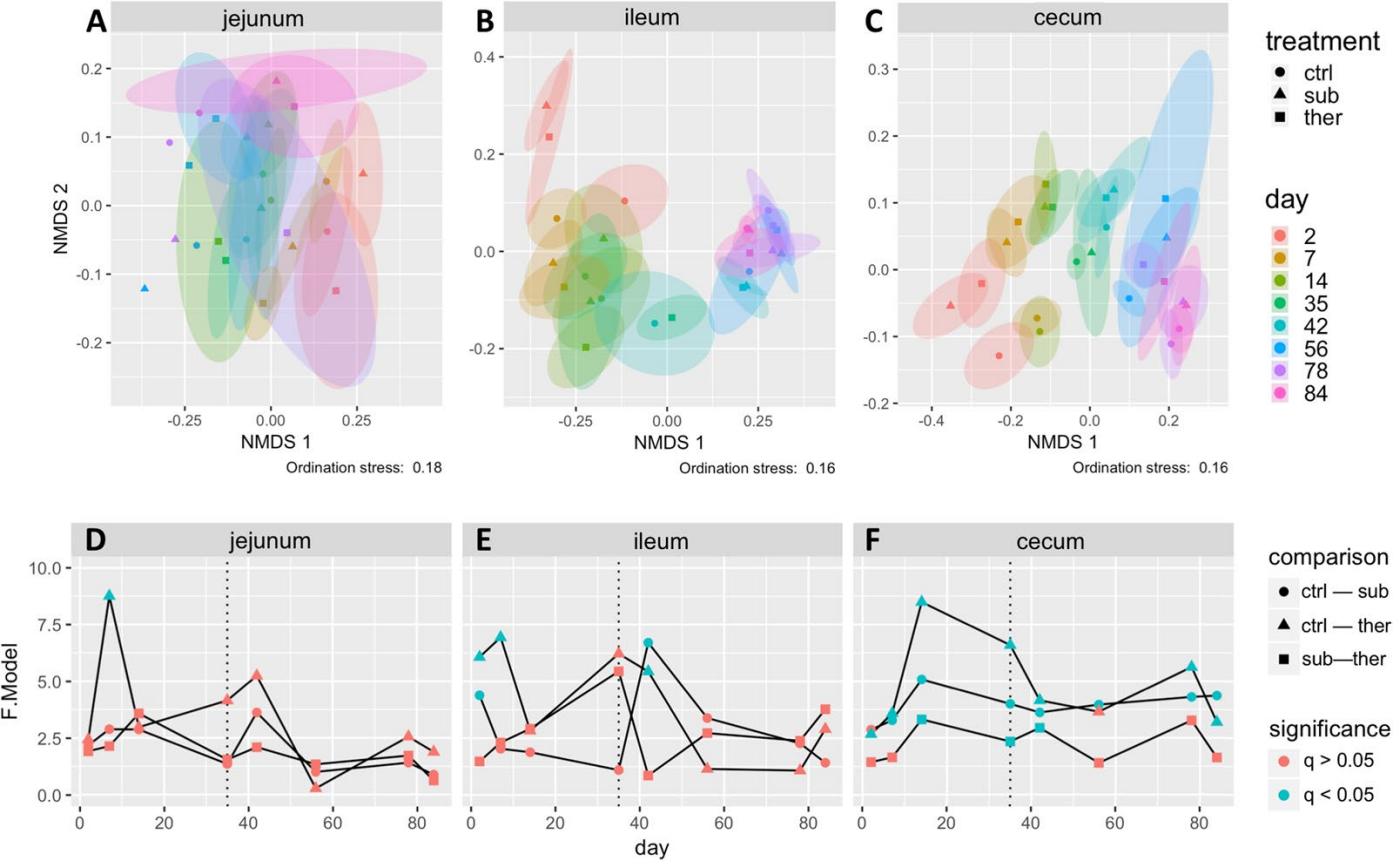
Beta diversity shifts of turkey cecal (**A**,**D**), ileal (**B**,**E**), and jejunal (**C**,**F**) microbiome through time and as affected by BMD treatment as estimated by the Bray-Curtis measure. A non-metric multidimensional scaling (NMDS) ordination was calculated (Fig. S1) for each gut location (indicated at top of each figure) individually (**A**–**C**) with stress indicated below each figure. Ellipses indicate the 95% confidence interval with the symbol at the centroid of all replicate samples of a treatment group (indicated by symbol shape) on each day (indicated by color). Individual replicate data points are omitted for clarity but all data points are shown in Fig. S1. The cecal and ileal communities mature through the 12-week span of this experiment, while the jejunal community matures to a lesser extent. Statistical testing (PERMANOVA) for differences in beta diversity due to BMD treatment are indicated in (panels D,E). The F value indicates the degree of difference between the two communities compared. Symbol shapes indicate the pairwise treatment beta groups tested and symbol color indicates if the beta diversity was statistically different. P-values were adjusted by using the false discovery rate method for multiple comparisons. BMD treatment resulted in different cecal bacterial communities at every time point considered, but fewer differences were observed in the ileal bacterial communities, and nearly no differences were observed in the jejunal bacterial communities due to BMD treatment. ctrl, no antibiotic control; sub, subtherapeutic BMD dose; ther, therapeutic BMD dose.

Microbial community structural differences as determined by the Bray-Curtis measure demonstrated that gut location had the strongest influence on the turkey microbial community, followed by age and antibiotic treatment (Fig. S1). Nonetheless, supplementing feed with BMD statistically altered (PERMANOVA, q < 0.05) bacterial composition in the ceca (throughout life of the turkey) when comparing time-matched treatment groups, but had less impact on the bacterial composition in the jejunum and ileum (Fig. 2). The bacterial community shifted less over time in the jejunum than in the ileum or the ceca.

The shifts in microbiota structure were BMD dose-dependent. During days 14–35 of the antibiotic treatment, the cecal communities of all three animal groups were statistically different (PERMANOVA, q-value < 0.05) from each other. One week following the BMD dose change in the therapeutic animals to a subtherapeutic dose, the communities of the two BMD groups remained different, despite receiving the same dose of BMD. However, after day 42 the bacterial communities from subtherapeutically and therapeutically treated animals were no longer statistically different (days 56–84) (Fig. 2D). Similarly, one week after withdrawal of BMD from feed, the microbial communities remained distinct between the BMD groups and the no antibiotic control group. The magnitude of the difference between the subtherapeutic and therapeutic groups in the ileum (PERMANOVA F statistic) also decreased after its highest value on day 35 (Fig. 2E). There was little impact of BMD on the jejunal microbial community. It appears clear that both in-feed BMD concentrations significantly altered the structure of the cecal microbiomes, dependent on dose.

**Figure 3:**
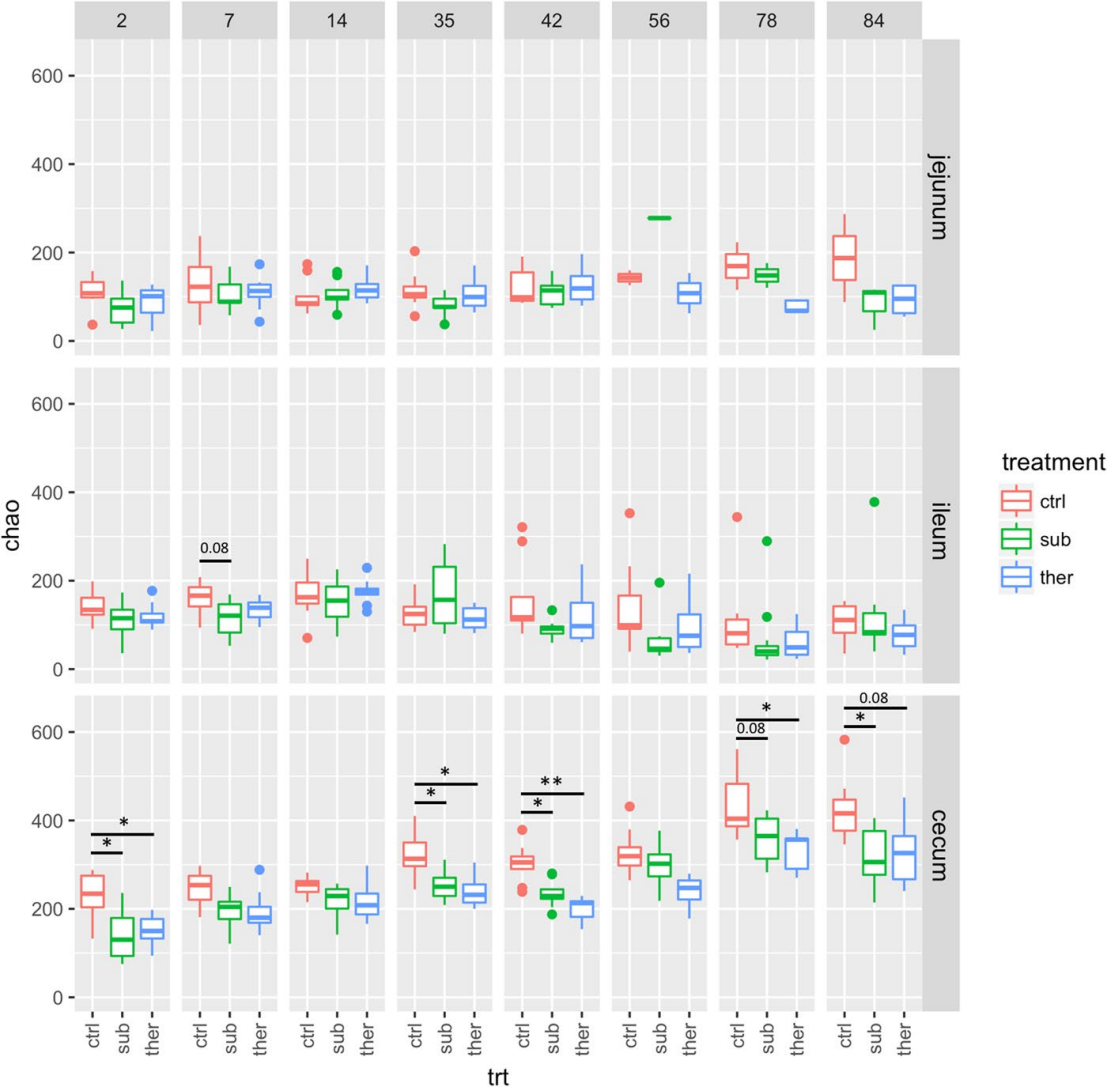
Alpha diversity of the turkey ceca through time and as affected by BMD treatment as estimated by the Chao measure of OTU richness. The number of OTUs increased about 2-fold as the turkeys matured from day two until day 84 of the study. BMD trended to reduce species richness throughout the experiment, with statistical significance (Tukey test) as indicated. P-values were adjusted by using the false discovery rate method for multiple comparisons. *0.05 > q > 0.01; **0.01 > q > 0.001. Panel upper labels indicate days after start of BMD diets. ctrl, no antibiotic control; sub, subtherapeutic BMD dose; ther, therapeutic BMD dose.

In addition to altering the bacterial beta diversity, BMD treatment reduced alpha diversity in the turkey ceca. In the control animals, there were approximately 100–200 OTUs (as predicted by the Chao index) throughout life in the turkey jejunum and ileum, while in the ceca there were about 250 OTUs early in life after which richness steadily increased to about 400 OTUs. Although neither dose of BMD altered OTU richness in the jejunum or ileum, richness was significantly decreased (q < 0.05) by about 100 OTUs in the ceca throughout the life of the turkey by both subtherapeutic and therapeutic doses of BMD (Fig. 3). These data suggest the loss of BMD susceptible populations. Other antibiotics similarly reduce bacterial richness in poultry, suggesting that a suppressed bacterial load is a conserved outcome of antibiotic therapy^17^. The Shannon index (another measure of alpha diversity accounting for richness and evenness) showed fewer differences than richness measures in the ceca (Fig. S2) indicating that microbiome evenness was less affected by BMD. The most abundant taxa (about 1% of the community in control turkeys) no longer detected after day 35 in BMD-treated animals was unclassified *Candidatus Saccharibacteria* (previously known as TM7) (Fig. S3). Members of the *Candidatus Saccharibacteria* phylum are obligate epibionts of specific *Actinobacteria* species^18^ so the loss of this taxa with BMD may be due to direct effects on this taxon, or the loss of their microbial host. Overall, the loss of less abundant OTUs contributed most to the decreased bacterial richness.

Decreased bacterial richness is a commonly observed response to a variety of in-feed antibiotics in poultry^19^ and swine^18,20^, but the impact of microbiota richness on animal growth and feed efficiency is only beginning to be studied. In cows, reduced bacterial richness in the rumen is correlated with increased feed efficiency^2^. Xu *et al*. found higher bacterial diversity in the ceca and lower body weights in free range compared to caged chickens^21^. Increased zinc administration, which can promote animal growth, also decreases bacterial diversity^22^. Thus, it seems feasible that decrease bacterial diversity in the gastrointestinal tract promotes animal growth, and this hypothesis merits further study.

**Figure 4:**
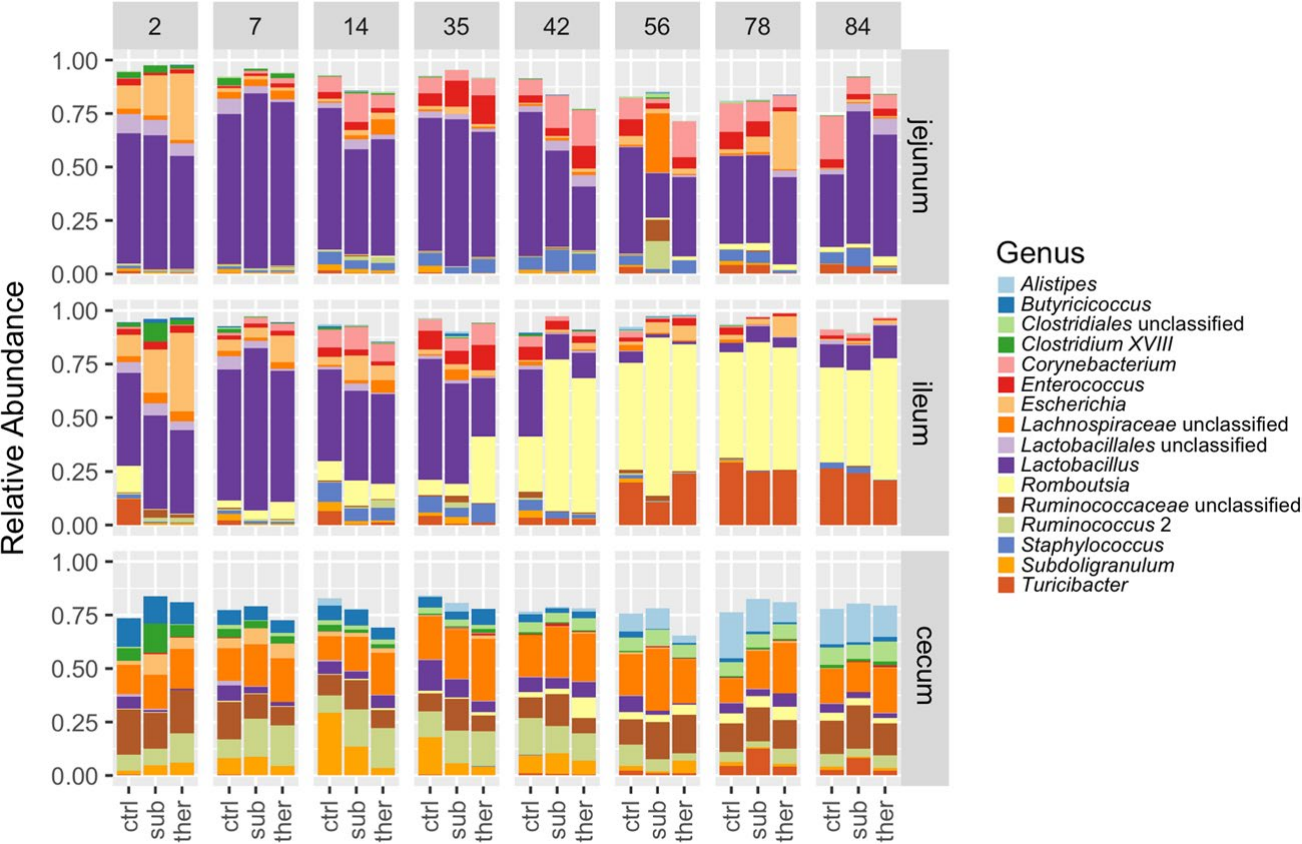
Bacterial composition of the turkey jejunum, ileum and ceca as indicated by genera that compose an average of >1% of the community in all gut locations. Other minor genera are not shown. All gut locations are dominated by genera from the phylum *Firmicutes*. Jejunal and ileal communities are dominated by *Lactobacillus*. This microbiota change, or maturation, through time was influenced but generally unimpeded by addition of BMD to the feed. There was a dramatic switch in the ileal community from being dominated by *Lactobacillus* at young ages to being dominated by *Romboutsia* after day 42. Specific statistical differences in genus composition are shown in Fig. S8. Panel upper labels indicate days after start of BMD diets. ctrl, no antibiotic control; sub, subtherapeutic BMD dose; ther, therapeutic BMD dose.

Bacterial membership of the intestinal tract differed according to intestinal location. Throughout the growth of the turkeys, the microbiotas were dominated by the phylum *Firmicutes* in the jejunum, ileum, and ceca, while *Proteobacteria* was found at low numbers in all gut locations. Other less abundant phyla differed between gut compartments. *Actinobacteria* mainly restricted to the jejunum, while *Bacteroidetes* was restricted to the ceca (Fig. S4). The jejunum was dominated by *Lactobacillus spp*. The ileum was dominated by *Lactobacillus* until seven weeks of age, while *Romboutsia* and *Turicibacter* composed about 70% of the ileal community after 10 weeks of age (Fig. 4). This shift, or maturation, occurred first in animals in the therapeutic BMD group, followed by the subtherapeutic and control animals, and agrees with other studies that have showed that gut microbiota succession occurs earlier in antibiotic treated animals^23^. The cecal community was dominated by members of *Lachnospiraceae* and *Ruminococcaceae* families regardless of treatment or time (Fig. S5). Thus, each intestinal segment contains distinct microbial communities.

Another proposed mechanism by which antibiotics may promote animal feed efficiency is selection of beneficial bacteria and/or inhibition or elimination of harmful populations. In our study, the most abundant genera that were enriched in the ceca early after BMD introduction were *Ruminococcus* 2, *Clostridium* XlVa, and *Fusicatenibacter*, and each are members of the *Lachnospiraceae* family. *Lachnospiraceae* spp. produce short chain fatty acids (SCFA) and have been enriched in chickens with improved feed efficiency^24,25^. This observation supports the possible antibiotic selection of microbiota with beneficial effects. Additionally, decreased abundance of members of the phylum *Candidatus Saccharibacteria* (TM7) due to BMD, as mentioned above, was potentially a beneficial impact, as they have been associated with inflammation and irritable bowel syndrome in humans^26^. Increased production of SCFA and decreased inflammation in poultry, would likely lead to decreased metabolic costs to the immune system and increase growth as a result^27^.

However, there were potentially beneficial bacteria that were reduced in abundance, especially soon after BMD treatment. *Lactobacillus* is a genus often used in growth promoting applications^25,28,29^, however relative abundance of *Lactobacillus* was decreased in the ceca of both BMD treatment groups on days 2–7 and the ileum on day 35. *Turicibacter* was decreased in the ileum on days 2–14 and *Subdoligranulum* was decreased between days 14–35 due to both subtherapeutic and therapeutic BMD. Both *Turicibacter*^30^ and *Subdoligranulum*^31^ are speculated to be beneficial bacteria associated with high fiber diet in broiler chickens. By day 78, the subtherapeutic group had higher abundance of *Turicibacter* in the ceca than the other groups (details illustrated in Fig. S4). In addition, similar to previous studies of other growth promoting antibiotics^10,12,18^, there was an increased relative abundance of *Escherichia* in the ceca two days following subtherapeutic BMD, which recovered by day 7. Given the remainder of the complex microbial community, we cannot determine the impact of these potential beneficial or harmful genera on the turkeys in our experiment. But we observe that the use of BMD enriches and inhibits both potentially beneficial and deleterious bacteria.

### Dose-dependent metabolome responses

**Figure 5:**
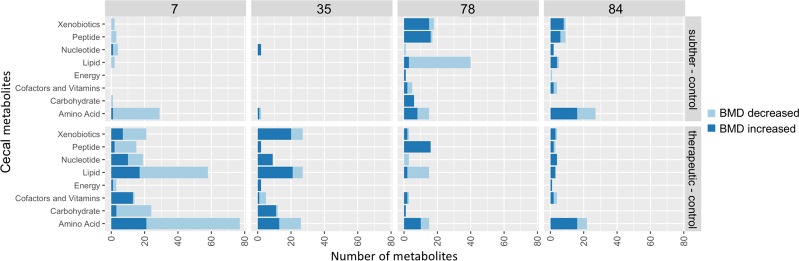
Counts of cecal metabolites that were differentially (q < 0.1) present in the animals given subtherapeutic (upper panel) or therapeutic (lower panel) in feed BMD compared to the control animals. Similar trends were generally observed in the therapeutic and subtherapeutic groups but to a lesser extent in subtherapeutic animals. Panel upper labels indicate days after start of BMD diets.

Global metabolomics revealed over 700 metabolites in the turkey ceca (524 metabolites were present across all groups and timepoints), and BMD treatment was correlated with changes in metabolites. Bacitracin concentrations acted as a proxy internal control in the experiment, because they were increased in the ceca, according to BMD treatment (Fig. S6). The dose-dependent responses to BMD, as observed in the microbiota data (Fig. 2) were also apparent in the metabolomic data. The cecal metabolite profiles of turkeys fed subtherapeutic and therapeutic BMD were statistically different (q < 0.05) on day 7 and day 35 (Fig. S7). However, after the therapeutic diet group transitioned to a subtherapeutic BMD concentration, the metabolite content of the therapeutic and subtherapeutic groups converged throughout the remainder of the experiment, but still different from the control (Figs 5 and S7). Of the metabolites present in all groups over the course of the experiment, 363/524 metabolites were significantly (q < 0.1) differentially present due to BMD treatment in at least one time point during the study, suggesting global impacts on microbial functions. Taken together, BMD exerted significant short- and long-term effects on both the microbiota and the metabolomic profile of the ceca and a period of recovery to homeostasis in the weeks that follow.

### BMD affected metabolite profile changed over time

After seven days, 44% (231/524) of all metabolites present at this timepoint were differentially abundant (ANOVA, q < 0.1) due to therapeutic BMD. Of these 68% (157) of the 231 differentially abundant metabolites were decreased due to therapeutic BMD, mainly related to host energy/nutrient sources, including amino acids, carbohydrates, lipids, nucleotides, peptides, and plant/food metabolites. Subtherapeutic BMD had a similar impact, but on fewer metabolites (41 metabolites differentially present, of these 39 were depleted due to BMD). Importantly, decreased cecal concentration of dietary amino acids, largely proteinogenic amino acids, at day 7 was evident in animals fed either concentration of BMD. However, by day 35, only 28% of the 110 differentially present metabolites in animals fed therapeutic BMD were less abundant with BMD, the opposite trend as observed on day 7. There were only 4 differentially present metabolites in the animals fed subtherapeutic BMD. One example of the change in metabolite profile at different time points were dipeptides, markers of protein metabolism that have a role in bacterial-host interactions^32^. On day 7, all (17) dipeptides trended to be less abundant (up to 3-fold reduced) in BMD-fed poults. But on day 78, the inverse relationship was observed, as 16 of the 17 dipeptides measured were increased, in both the subtherapeutic (2-4-fold increased) and therapeutic (2-7.5-fold increased) BMD groups compared to the no antibiotic control group (Fig. S9).

The impact of the shift from decreased (early) to later increased metabolite concentration is unclear, but may be consistent with or due to the consequences of early bacterial inhibition and later bacterial recovery to a new stable state. Some metabolite shifts detected in the experiment suggest bacterial inhibition early in the experiment. On day 7, adenine metabolites were strongly impacted by BMD. Adenosine triphosphate (ATP) and deoxy-ATP precursors, adenosine 5′-monophosphate (AMP), adenosine 5′-diphosphate (ADP), and 2′-deoxyadenosine 5′-monophosphate (dAMP), respectively, increased in abundance due to BMD, while some precursors (adenine, adenosine, 1-methyladenine, 2′-deoxyadenosine) were less abundant. Changes to nucleotide abundances seemed to be short-term, primarily on day 7. Importantly, increases in adenosine nucleotides in bacteria, especially ATP, are linked to bacteriostatic antibiotics^33–35^. Additionally, nearly all phospholipids were decreased (as much as 50-fold) due to therapeutic BMD on day 7 and decreased phospholipids, a major component of gram-positive and gram-negative cell membranes^36^, may indicate an overall reduction of intestinal bacterial load. Similar observations of microbiota disturbance followed by microbiota recovery have been observed as a collateral effect in other antibiotic trials, but the impact of the phenomenon on the metabolome has not been adequately explored^10,18,37^.

### Amino acid concentrations were broadly impacted by in-feed BMD

In addition to BMD effects to protein metabolism manifested by changes in dipeptides, amino acid metabolite concentrations were significantly altered in turkey cecal contents with either concentration of in-feed BMD and were the most impacted category of metabolites in the experiment. Seven days following introduction of BMD, more amino acids and related derivatives were differentially present (ANOVA, q < 0.1) than any other metabolite category, and the majority of the amino acids and derivatives were less abundant due to BMD. Of the 20 basic proteinogenic amino acids, 19 were statistically or trended to be less abundant in both the therapeutic and subtherapeutic groups. Glutamate was unchanged (1.02 fold-change) in the therapeutic group compared to the control group. Other amino acid derivatives were also generally less abundant due to BMD. Additionally, according to random forest analysis of day 7 results, 21 of 29 metabolites that strongly distinguished either of the BMD treatment groups from the control group (Fig. S9) were amino acid metabolites, of which six were proteinogenic amino acids. Three amino acid metabolites (tryptophan, tyramine, and valine) distinguished both BMD-treatment groups from the control group (Fig. S9). Tryptophan metabolites were among the compounds most predictive of BMD treatment (according to random forest analysis) in both the subtherapeutic and therapeutic groups at days 35, 78 and 84. Changes were detected in indole-3-carboxylic acid (3 to 12-fold decreased after BMD treatment, days 35–84), 5-hydroxyindoleacetate (2-fold decreased due to therapeutic BMD, day 35, 9-fold enriched in both BMD groups, day 84), indoleacetate (7 to 13 fold enriched in both BMD groups, day 84), and N-acetylserotonin (2-fold decreased due to therapeutic BMD only, day 78) (Fig. S9 and Table S2). Tryptophan metabolites are a long-responding group of metabolites to both doses of BMD. Gadde *et al*. also found that amino acids in the ileum were significantly impacted by BMD, generally increased in that gut compartment in chickens at 21 days of age fed BMD since day of hatch^13^, indicating that altered amino acid metabolism is a common result of BMD treatment in poultry.

### Metabolome-microbiome interactions

A computational approach was used to find correlations between OTU abundance patterns and metabolite levels. Inferred bacterial species abundance patterns were correlated with more metabolite concentrations (especially amino acids and nucleotides) in the control group (30 metabolites) than in either group given BMD (18 and 23 metabolites in the subtherapeutic and therapeutic groups, respectively) indicating that microbial metabolic pathways were likely disrupted by in-feed BMD (Fig. S10). Furthermore, in the control animals, 27 species were correlated with 3 or more metabolites; however, only 5 and 6 species were associated with 3 or more metabolites in the subtherapeutic and therapeutic animals, respectively (Fig. S11). Genera whose abundance were decreased by BMD either early or late in the experiment generally correlated with the abundance of fewer numbers metabolites in BMD treated animals. For example, unclassified *Firmicutes*, unclassified *Candidatus Saccharibacteria*, *Subdoligranulum*, *Lactobacillus brevis*, *Spirochaetes*, *Lactobacillus plantarum*, *Butyricicoccus*, *Dorea* and *Akkermansia* all correlated with the abundance of 5 or more metabolites in the control animals but 2 or fewer (often 0) in the BMD-treated animals (Fig. S9). On the other hand, two members of genera that were enriched with BMD, *Clostridium clostridioforme* and *Ruminococcus torques* were correlated with more metabolites in the therapeutic group than in the control group (Fig. S11) indicating the potential impacts of these two genera in the therapeutic animals. Using an alternative method, a weighted correlation network analysis (WGCNA)^38^, it was observed that correlations between metabolites and bacterial clusters were rarely the same between treatments (data not shown), which supports the findings using the MIMOSA method that metabolic pathways were perturbed by BMD. Taken together, it appears that bacterial composition predicts metabolome composition but normal metabolic networks are perturbed in animals fed BMD. Recent metabolomic studies indicate that antibiotics alter the respiration and metabolic state of bacteria^34,39,40^, and the metabolic response is dependent on the mechanism of action of the antibiotic^41^. In addition, the antibiotic carbadox, given subtherapeutically, impacts expression of bacterial metabolism genes in swine^12^. More investigation is warranted on the specific impacts of growth promoting antibiotics on the metabolic pathways of intestinal bacterial populations by shotgun metagenomics or metaproteomics.

**Figure 6:**
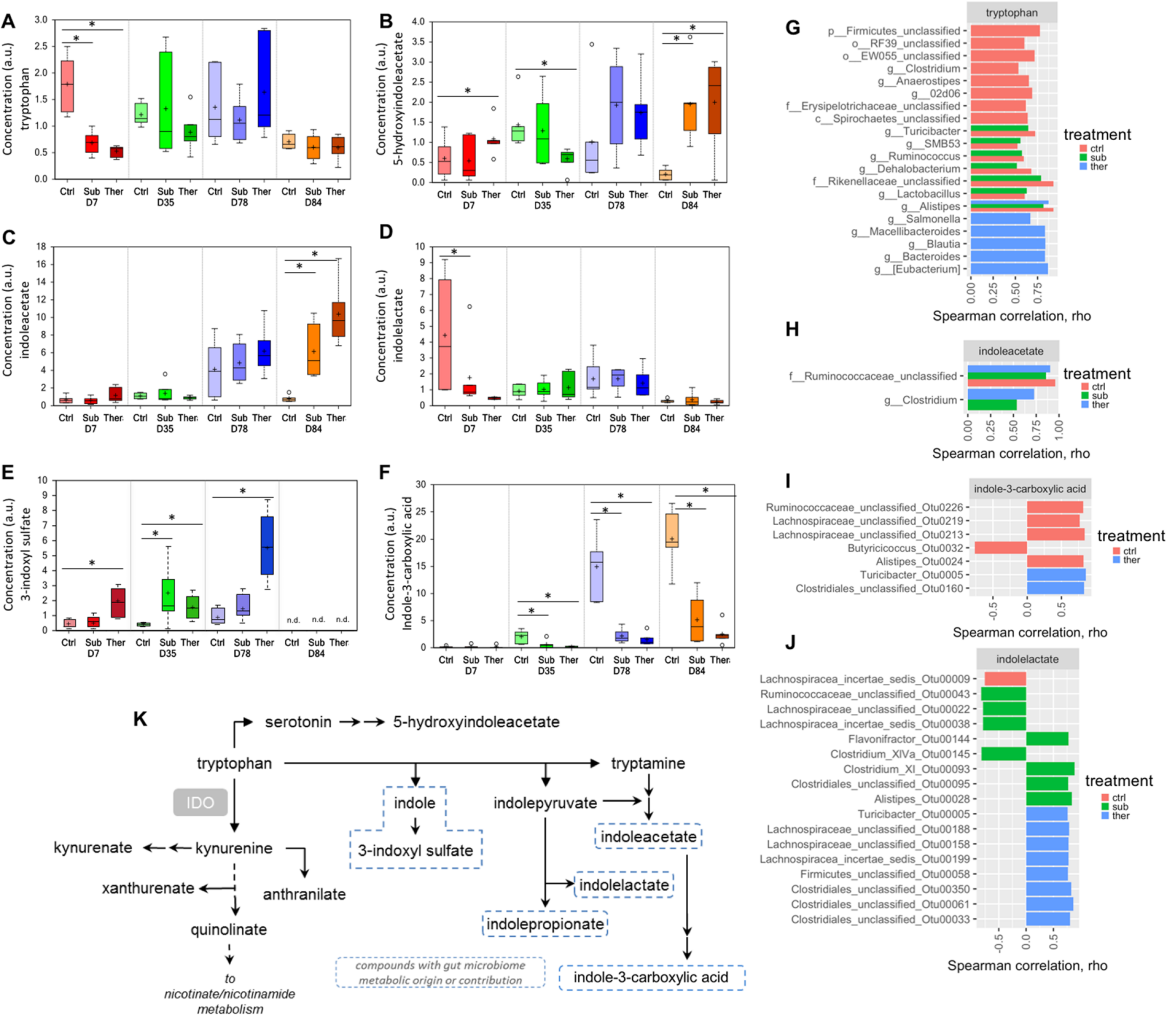
The differential presence of tryptophan-derived metabolites (**A**–**F**) and the bacterial taxa correlated with metabolite (indicated in panel upper label) concentration (**G**–**J**) in each treatment group. Many tryptophan-derived metabolites were differentially present due to BMD treatment. The bacterial taxa with significant correlation with these metabolites (q value < 0.05, rho >0.5) were generally distinct between the control and therapeutic groups, while there was some overlap between the subtherapeutic group and the other two treatment groups. ctrl, no antibiotic control; sub, subtherapeutic BMD dose; ther, therapeutic BMD dose. The tryptophan degradation pathway (K) is provided for reference.

Tryptophan metabolites were strongly predictive of BMD use (Fig. S9) and we investigated microbiota correlated with their abundance to gain greater insight to their metabolism. Tryptophan is a precursor for a large number of microbial- and host-metabolites^42^. The majority of dietary tryptophan^43^ is metabolized in the intestinal tract by microorganisms into indole and indole-metabolites indole-3-aldehyde, indole-3-acetic acid (IAA), indole-3-propionic acid (IPA), indole-3-acetaldehyde and indoleacrylic acid. These microbiota-derived metabolites are endogenous ligands for the aryl hydrocarbon receptor (AhR), which regulates immune response and homeostasis at the level of intestinal epithelium. Intestinal contents from germ-free mice fail to induce AhR signaling, demonstrating the role of microbiota in producing these ligands^5,44,45^. We measured a number of tryptophan metabolites including indole molecules (Fig. 6) and indole-3-carboxylic acid, indoleacetate (conjugate base of IAA), and 5-hydroxyindoleacetate were among the top metabolites distinguishing both BMD doses from the control birds (Fig. S9). To date, only a few specific bacteria are known to produce endogenous ligands of the AhR, which affected host-physiology. In our dataset, we identified bacterial taxa correlated with tryptophan abundance and three families compose most of the species correlated with tryptophan metabolite concentrations, including *Clostridiales*, *Lachnospiraceae*, and *Ruminococcaceae* (Fig. 6H–J). This finding agrees with previous data, where *Clostridium sporogenes*, a member of *Clostridiales*, metabolized tryptophan to IAA and IPA, both molecules modulate intestinal permeability and immunity^45–47^. Additionally, in our dataset different bacterial populations correlated with tryptophan metabolite concentration depending on BMD treatment. For example, the species correlated with tryptophan levels in the control and therapeutic groups were not the same, while some strains correlated with tryptophan levels in the control group were also predictive in the subtherapeutic group. This pattern was similar with IAA or indoleacetate. However, species correlated with indole-3-carboxylic acid and indoleacetate were mutually exclusive to treatment group. Furthermore, the bacterial functional redundancy (or number of species correlated with metabolite abundance) differs by metabolite with more functional redundancy with tryptophan in the control group, but more redundancy for indolelactate in both groups of BMD treated animals. In the ceca, BMD increased the amount of IAA, 3-indoxyl sulfate, and 5-hydroxyindoleacetate as well as decreased indole-3-carboxylic acid at the end of the experiment (days 78–84). In humans with inflammatory bowel disease, there is inverse correlation of tryptophan metabolism and production of endogenous AhR ligands and severity of clinical disease. For example, humans with ulcerative colitis have less IPA in their serum compared to healthy age-matched patients^48^. Thus, increased levels of IAA and 3-indoxyl sulfate may have a beneficial effect. Future studies are needed to better determine whether BMD-induced tryptophan metabolites promote the health of turkeys.

## Conclusions

Our findings indicate in-feed BMD impacted intestinal bacterial membership and function, and the magnitude was dose dependent. Additionally, we found that the microbiota composition correlated with the metabolomic composition and that populations that were enriched in one treatment group were correlated to more metabolites than in other treatment groups. Thus, it seems apparent that microbiota shifts are driving metabolite shifts. The coupling of metabolomics with bacterial membership surveys improves our understanding of the microbiota functional output, and the compounds interacting with the host. Microbial taxa associated with beneficial functions should be explored as potential targets for modulation to improve animal health and production.

## Materials and Methods

### Animal treatments and sample collection

This experiment was conducted according to the approval of the National Animal Disease Center (NADC) Institutional Animal Care and Use Committee (IACUC) protocol ARS-2869. Two-hundred and forty male day-of-hatch Nicolas turkey poults (Valley of the Moon Hatchery, Osceola, Iowa) were obtained and co-housed to acclimate for two weeks prior to the start of adding antibiotic to feed. Initially, all turkeys were housed together, in one room, on conditioned litter obtained from the NADC non-antibiotic treated specific-pathogen free Small-Beltsville white turkey flock, which is consistent with industry practices to homogenize microbiotas across birds^49^. The rooms were on a 12 hour light/dark cycle with positive air pressure (>10 air changes per hour). Turkeys were *ad libitum* fed a turkey starter ration the first seven weeks of life, followed by grower rations for eight weeks in all treatment groups. Diet formulations are provided (Table S1). At two weeks of age, poults were randomly divided into three treatment groups (no antibiotic control, subtherapeutic BMD (50 g/ton feed) or therapeutic BMD (200 g/ton)). Each treatment group was floor housed in one animal Biosafety Level 2 room. Subtherapeutic BMD was given continuously for 11 weeks. Therapeutic BMD was fed for five weeks, followed by reduction to subtherapeutic concentration for the next six weeks of the experiment. After 11 weeks of subtherapeutic or therapeutic BMD, antibiotic was withdrawn for 1 week before the end of the study. Ten turkeys from each treatment group were humanely euthanized starting as early as two days after feeding different BMD treatments (16 days post-hatch) until one week after withdrawal from BMD (Fig. 1). After euthanasia, cecal, ileal, and jejunal contents were sampled from each bird for microbiota analysis. Two hundred mg of cecal contents from each bird were flash frozen in liquid nitrogen for metabolome analysis^50^.

### Characterizing the antibiotic effects on the microbial community

Briefly, total DNA from intestinal contents were isolated by mechanical lysis (PowerMag Microbiome DNA/RNA isolation kit, MO-BIO laboratories, Carlsbad, CA). Bacterial 16S rRNA genes (V1–V3 region) were PCR amplified and sequenced on the MiSeq platform (Illumina Inc., San Diego, CA)^51^. Data were deposited in the NCBI Short Read Archive under the Bioproject PRJNA485957. 16S rRNA gene sequences were aligned, curated, and analyzed using the Mothur software package, following the Schloss MiSeq SOP^52^. Sequences that appeared only once (singletons) or twice (doubletons) across the all samples, were eliminated as previously described^53^. Measures of alpha diversity were calculated using mothur. Community beta diversity analysis was completed in R using the vegan package. Non-metric multidimensional scaling ordinations were calculated using the Bray-Curtis measure of beta diversity. PERMANOVA testing was completed using adonis function from the vegan package.

### Metabolomics analysis

Lyophilized cecal contents (approximately 200 mg wet weight) were sent to Metabolon Inc. (Durham, NC) for global metabolomic analysis using a non-targeted UPLC-MS/MS approach. A subset of samples (n = 72) were selected for metabolic profiling, and included six samples from each experimental group at four time points (day 7, 35, 78, 84). Methanol extractions were divided into fractions for different analyses: two separate reverse phase (RP)/UPLC-MS/MS methods with positive ion mode electrospray ionization (ESI), analysis by RP/UPLC-MS/MS with negative ion mode ESI, and analysis by HILIC/UPLC-MS/MS with negative ion mode ESI^17^. Compounds were identified by comparison to Metabolon’s library entries of purified standards, based on retention time and accurate mass ±10 ppm. Peaks were quantified by integrating the area under the curve. To normalize the data, each biochemical was rescaled to set the median equal to 1 and missing values were imputed with the minimum value as the detection limit. Following this method, values are not absolute concentrations but are accurate relative to each other. Samples were analyzed in two batches (batch 1: days 7, 35, 78 (Supplementary Data 1); batch 2 (to evaluate effects of antibiotic withdrawal): day 84 and the control group from day 78 (Supplementary Data 2)), with multiple samples from batch 1 to act as technical controls to ensure accurate measurements between batches. When the two batches were combined into a single data set (Supplementary Data 2), 524 metabolites were shared between both batches. To identify metabolites that were significantly different due to dietary treatment, analysis of variance (ANOVA) was used between time-matched samples following median scaling of the original data. The false discovery rate (FDR) correction was applied to all metabolite testing and a q-value < 0.1 was considered statistically significant. Random forest analysis was completed in Rstudio using the randomForest function from the randomForest package. All R scripts for both microbiota and metabolome analysis are available at https://github.com/john2929/bmd_turkey.

### Metabolome-microbiome interactions analysis

We investigated correlations between the microbiome and metabolome composition data sets to identify bacterial populations that might be responsible for metabolite patterns, and how correlations between metabolites and microbiome composition change due to BMD. A Mantel test, which tested the overall correlation of the metabolite concentrations and OTU abundances (mantel function, vegan package version 2.4–6, R software), showed the phylogenetic and metabolomic compositions were statistically significant and fairly predictive (p < 0.001, r = 0.45) of each other. To further investigate this relationship, we interrogated the data from days 7, 35 and 78 (pre-withdrawal), the unscaled metabolome dataset and reference-based OTU table, using direct spearman correlations as in Williams *et al*.^54^ or by using MIMOSA, a metabolic model framework that integrates metabolic potential from bacterial genomes and metabolome composition^55^ into a unified analysis. We followed the MIMOSA protocol for predicting metagenome content from taxonomic composition, which uses the best-matched bacterial species composition to predict the metabolic potential in the metagenome (using the PICRUSt software^56^) and the resulting predicted metagenomes were normalized using MUSiCC (release 1.0)^57^ for metabolites with metabolic pathways included in the Kyoto Encyclopedia of Genes and Genomes (KEGG) database. Then, using the MIMOSA software, metabolome profiles are predicted based on the microbial metabolic genes and compared to the actual metabolome data obtained experimentally.

## Supplementary information

Supplementary Information

Supplementary Data 2

Supplementary Data 1
