# Supplementary material for: In-feed bacitracin methylene disalicylate modulates the turkey microbiota and metabolome in a dose-dependent manner
Source: Sci Rep. 2019 Jun 3;9:8212. doi: 10.1038/s41598-019-44338-5 (PMC6547706; doi:10.1038/s41598-019-44338-5)
Supplement: Supplementary file 1 — Supplementary Information [file 41598_2019_44338_MOESM1_ESM.pdf]

**Supplementary Information:**

# In-feed bacitracin methylene disalicylate modulates the turkey microbiota and metabolome in a dose-dependent manner

Timothy A. Johnson[1,2], Matthew J. Sylte[1], Torey Looft[1*]

[1]National Animal Disease Center, Agricultural Research Service, United States Department of Agriculture, Ames, IA 50010, USA

[2]Current Address: Department of Animal Sciences, Purdue University, 270 S Russell St, West Lafayette, IN 47907

*Corresponding author:

National Animal Disease Center, P.O. Box 70, 1920 Dayton Ave., Ames, IA 50010

Telephone: 1-515-337-7140, E-mail: torey.looft@ars.usda.gov

**Table S1:** Turkey feed composition and nutritional content[1].

| | Turkey Starter | Turkey Grower |
|---|---|---|
| Ingredients | 0-7 weeks | 8-14 weeks |
| Yellow corn | 48.09 | 64.43 |
| Soybean meal (48.5%) | 46.94 | 31.85 |
| Ground limestone | 1.10 | 0.97 |
| Dicalcium $PO_4$ (18% P-22% Ca) | 2.48 | 1.50 |
| Micronutrients[2] | 0.50 | 0.50 |
| Salt | 0.36 | 0.33 |
| MHA Methionine | 0.18 | 0.05 |
| Vegetable oil | 0.30 | 0.32 |
| L-Lysine | 0.05 | 0.05 |
| | | |
| Calculated Analysis | | |
| Protein | 27.00 | 21.00 |
| Calcium | 1.10 | 0.80 |
| Non-plant phosphorus | 0.60 | 0.40 |
| Methionine | 0.58 | 0.38 |
| Methionine + cystine | 1.00 | 0.76 |
| Lysine | 1.55 | 1.15 |
| Tryptophan | 0.37 | 0.27 |
| Sodium | 0.16 | 0.15 |
| Chloride | 0.26 | 0.25 |
| Metabolizable energy (Kcal/lb) | 1270 | 1350 |

[1] Adapted from Damron, B.L., & Sloan, D.R. Poultry Diets for Small Flocks. IFAS Extension, University of Florida, SSPSE6, 1-3 (1995)

[2] Micronutrient mixture included: vitamin A, 3,000 IU; vitamin D3, 1,000 IU; vitamin E, 5 IU; menadione dimethylpyrimidinol bisulfite, 1 mg; riboflavin, 2 mg; pantothenic acid, 6 mg; niacin, 27.1 mg; choline chloride, 454 mg; vitamin B, 1.1 mcg; biotin, 0.05 mg; ethoxyquin, 0.0125%; manganese, 27.3 mg; iron, 22.7 mg; copper, 2.7 mg; cobalt, 0.09 mg; iodine, 0.5 mg; zinc, 27.3 mg

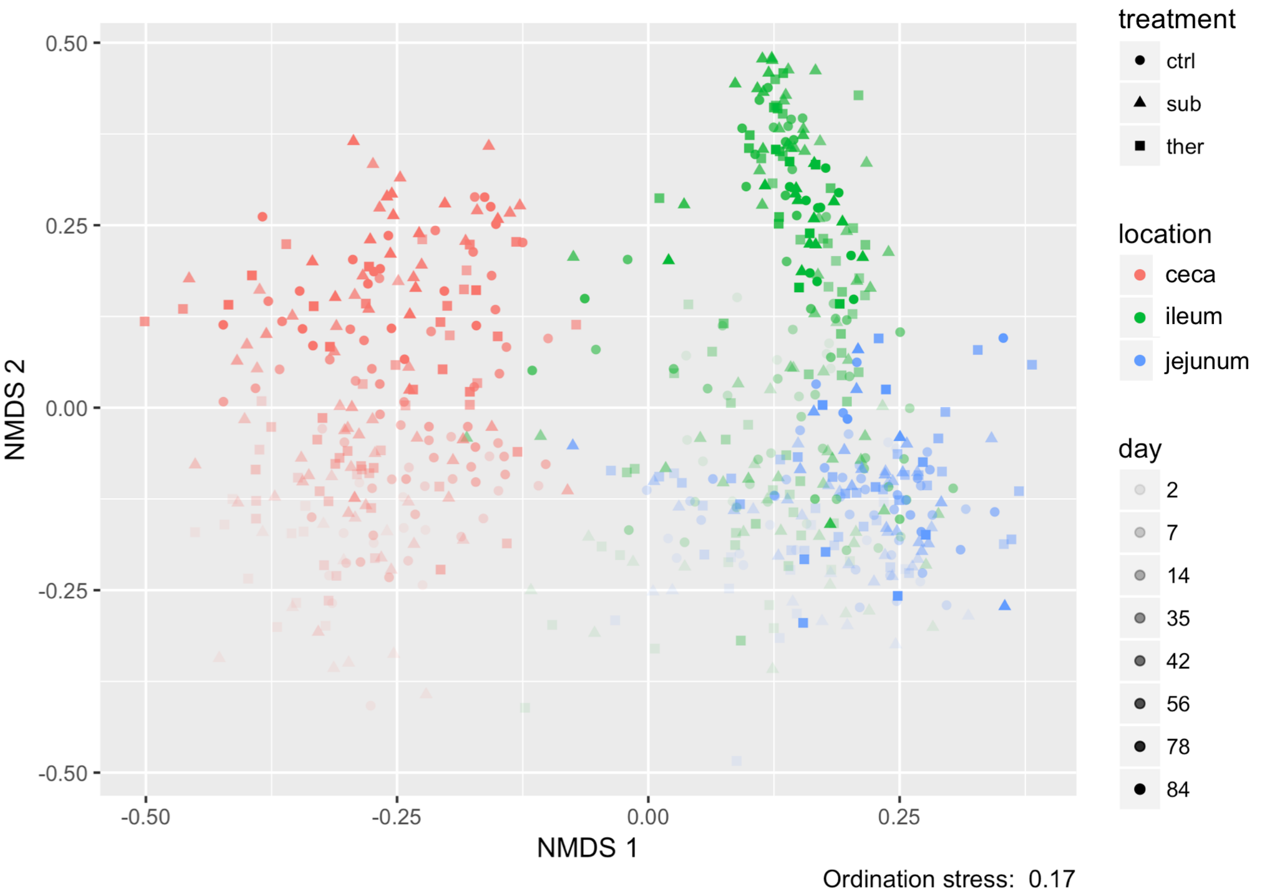


**Fig. S1:** Non-metric multidimensional scaling (NMDS) ordination for all data points (stress = 0.17). It is clear that the community structure of each gut location was distinct. All individual replicate data points are shown, whereas only the treatment group centroid was shown in Fig. 2. The cecal and ileal communities mature though the 2-14 week span of this experiment, while the jejunal community matures to a lesser extent. BMD treatment is indicated by shape, gut location is indicated by color, and day after BMD began is indicated by fill color transparency. ctrl, no antibiotic control; sub, subtherapeutic BMD dose; ther, therapeutic BMD dose.

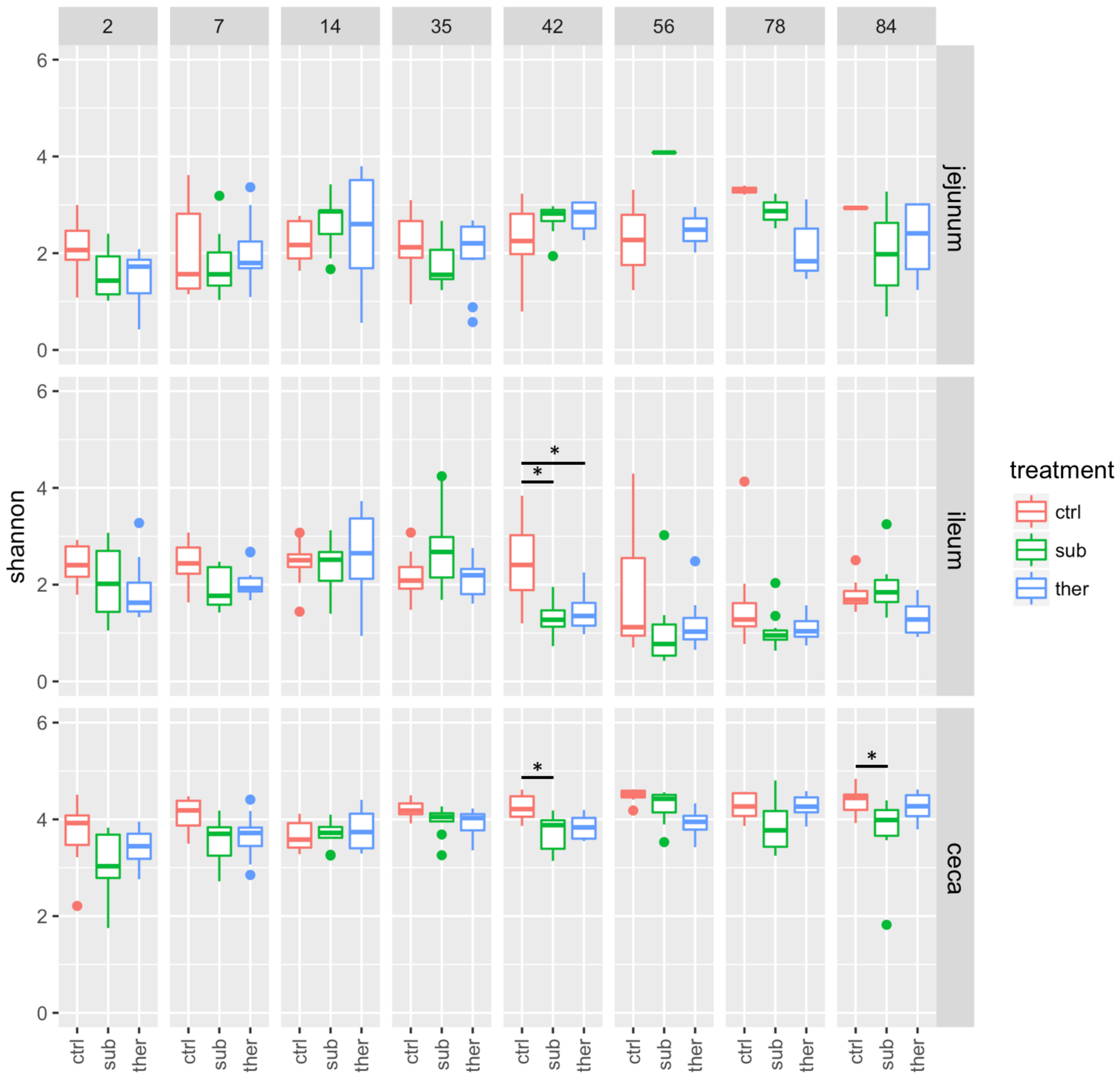


**Fig. S2:** Alpha diversity of the turkey ceca through time and as affected by BMD treatment as estimated by the Shannon measure of diversity. BMD trended to reduce Shannon diversity throughout the experiment (but to a lesser extent than bacterial richness). Statistically significant differences (Tukey test) are indicated. P-values were adjusted by using the false discovery rate method for multiple comparisons. * $0.05 > q > 0.01$; ** $0.01 > q > 0.001$. Panel upper labels indicate days after start of BMD diets. ctrl, no antibiotic control; sub, subtherapeutic BMD dose; ther, therapeutic BMD dose.

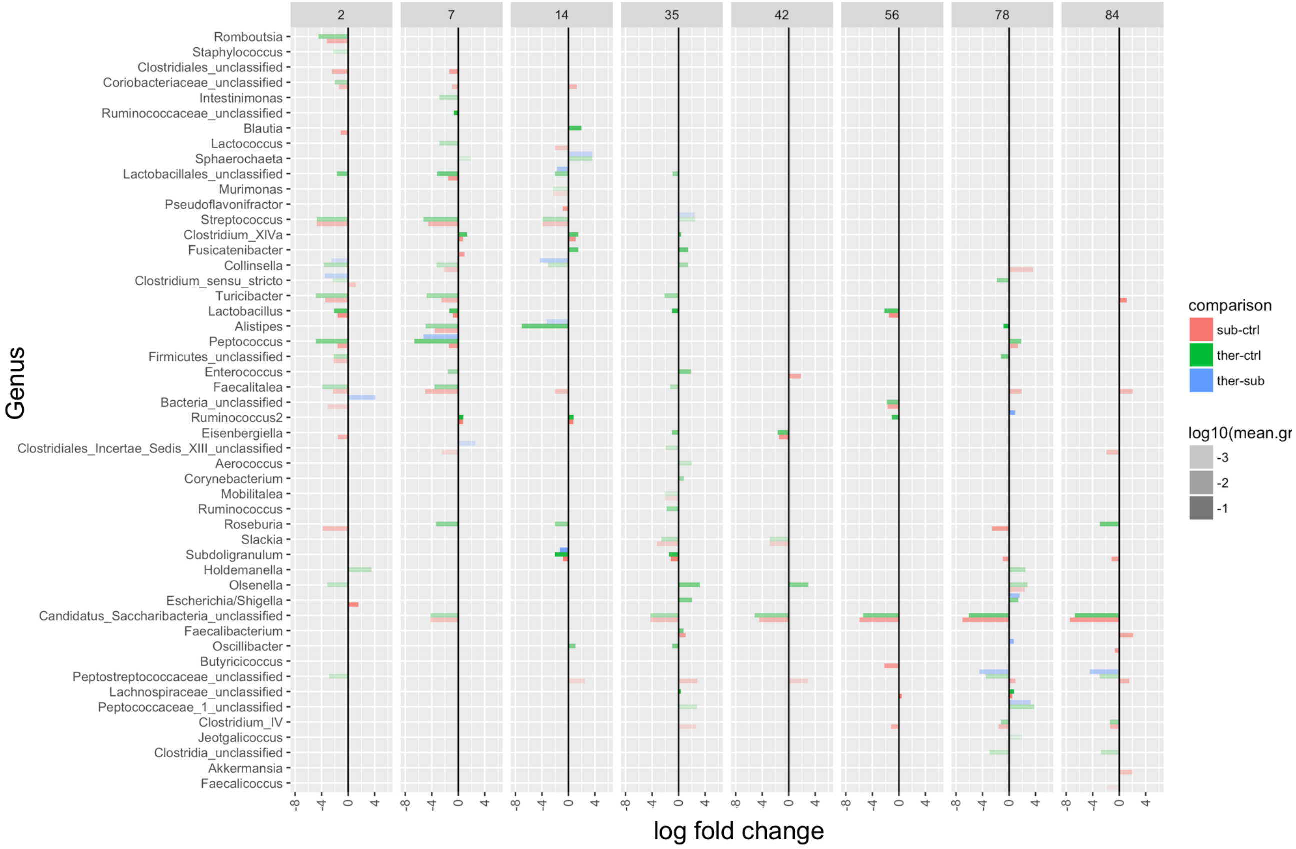


**Fig. S3:** Statistically differentially abundant genera ($q < 0.1$) due to treatment, based on phylotype-based sequence assignment. Fold change is expressed as $\log_{10}$ fold change, with negative values being decreased due to BMD and positive values being increased due to BMD. When the therapeutic and subtherapeutic groups are compared the values are relative to the subtherapeutic group. Genera are sorted by the day when their abundance was the greatest. Fill intensity decreases according to the value of the sum of the abundance of both groups considered. Panel upper labels indicate days after start of BMD diets. ctrl, no antibiotic control; sub, subtherapeutic BMD dose; ther, therapeutic BMD dose.

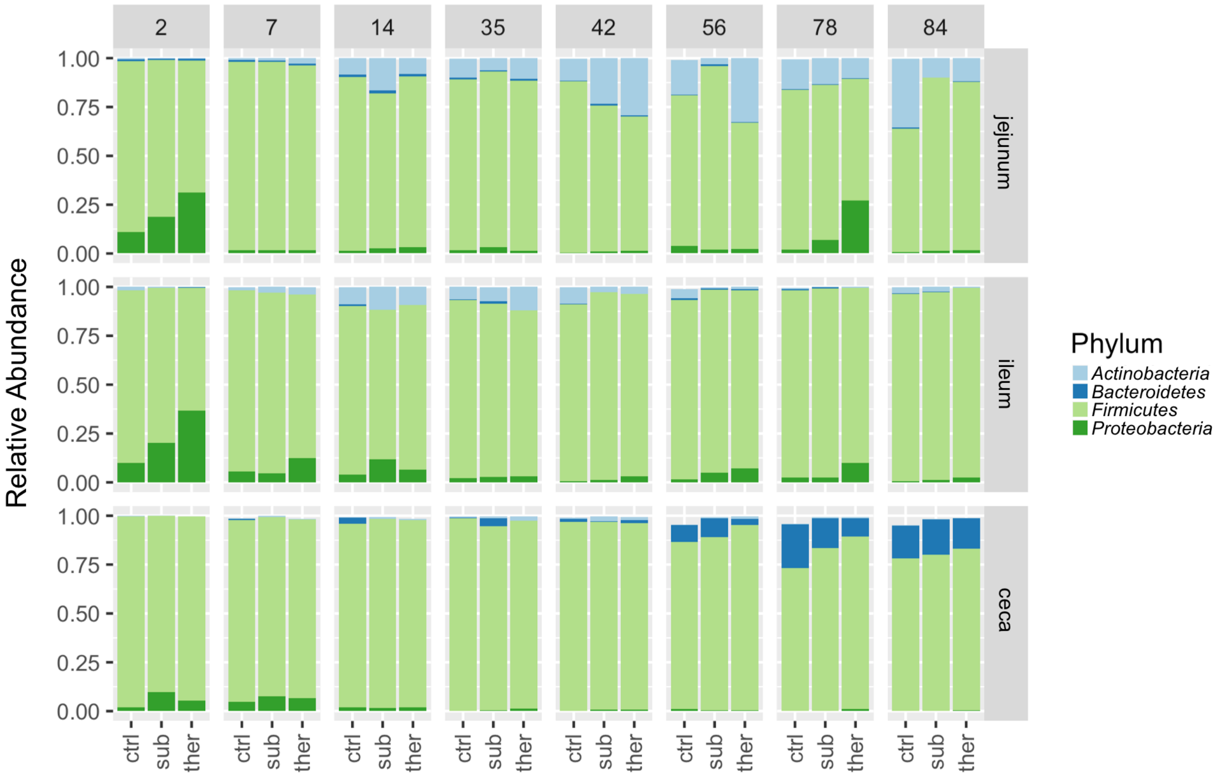


**Fig. S4:** Bacterial composition of the turkey jejunum, ileum and ceca as indicated by phyla that compose an average of >1% of the community in all gut locations. Other minor phyla are not shown. All gut locations are dominated by genera from the phylum *Firmicutes. Actinobacteria* composes up to 25%, 10%, and 1% of the bacterial community in the jejunum, ileum, and ceca, respectively, while *Bacteroidetes* was largely only detected in the ceca. Panel upper labels indicate days after start of BMD diets. ctrl, no antibiotic control; sub, subtherapeutic BMD dose; ther, therapeutic BMD dose; jc, jejenum contents; ic, ileal contents; cc, cecal contents.

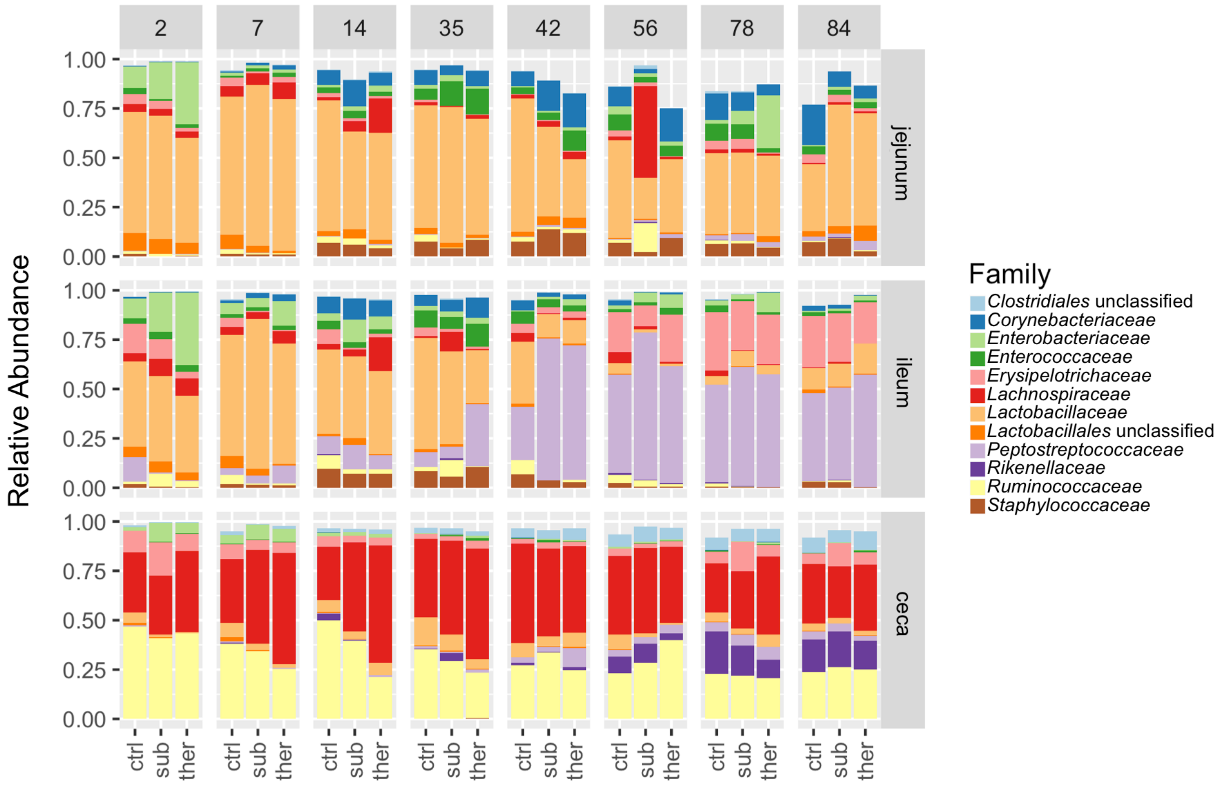


**Fig. S5:** Bacterial composition of the turkey jejunum, ileum and ceca as indicated by taxonomic families that compose an average of >1% of the community in all gut locations. Panel upper labels indicate days after start of BMD diets. ctrl, no antibiotic control; sub, subtherapeutic BMD dose; ther, therapeutic BMD dose; jc, jejenum contents; ic, ileal contents; cc, cecal contents.

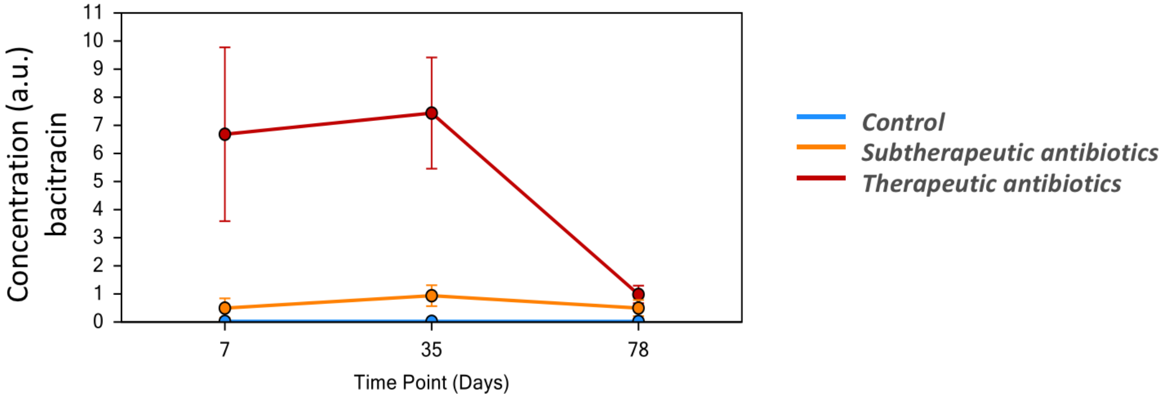


**Fig. S6:** Relative concentration of bacitracin in the turkey ceca in the three treatment groups at the different time points indicated. The therapeutic group had more bacitracin detected than was detected in the subtherapeutic group until the BMD dose changed after day 35.

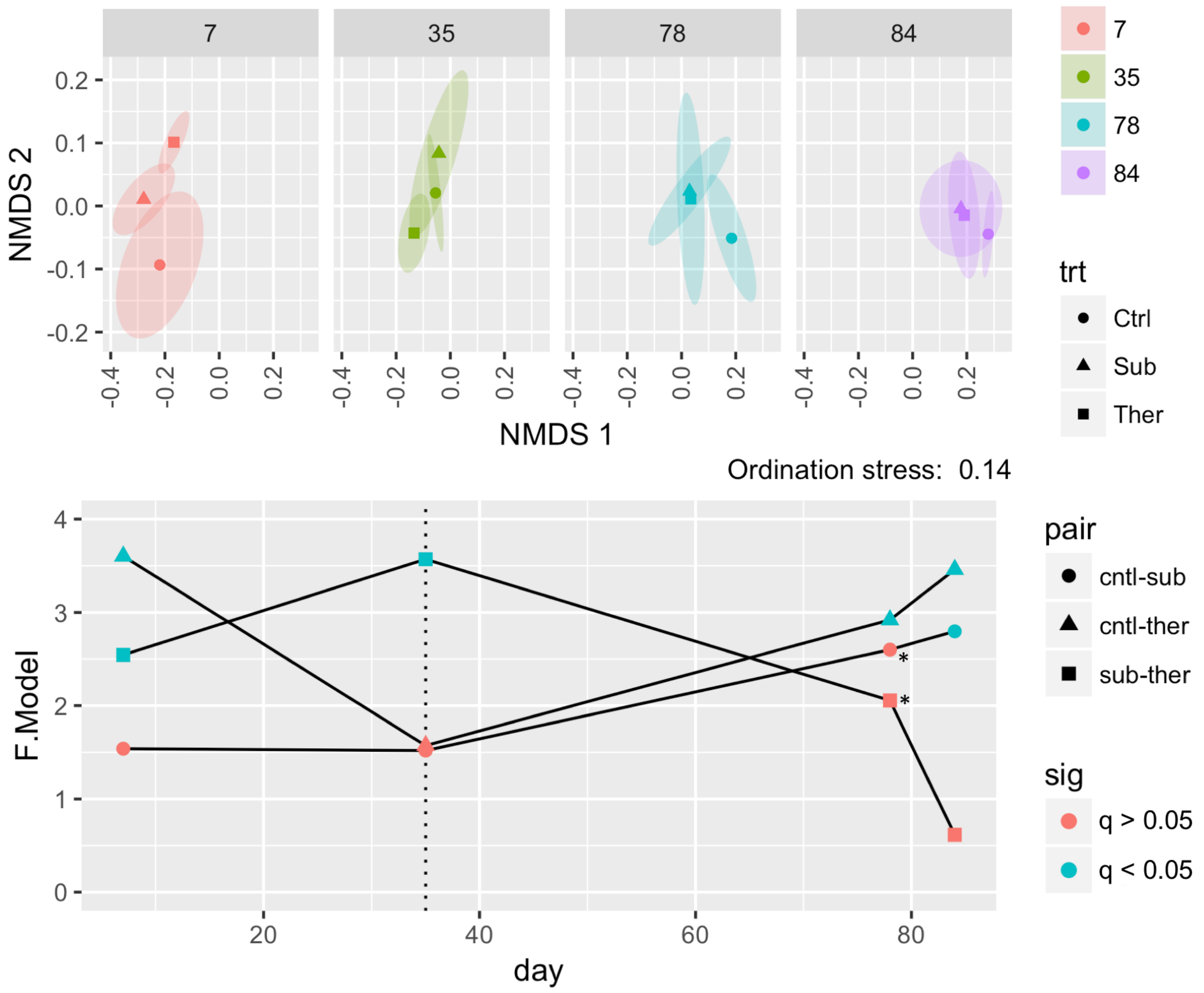


**Fig. S7:** Beta diversity shifts of turkey cecal metabolome through time and as affected by BMD treatment as estimated by the Bray-Curtis measure. A single non-metric multidimensional scaling (NMDS) ordination was calculated for all data points (stress = 0.14), even though each time point is shown in a separate panel (A-D) for clarity. It is clear that the metabolome profile evolves over time. Ellipses indicate the 95% confidence interval with the symbol at the centroid of all replicate samples of a treatment group (indicated by symbol shape) on each day (indicated by color). Individual replicate data points are omitted for clarity. Statistical testing for differences in beta diversity due to BMD treatment are indicated in panel F. Symbol shapes indicate the pairwise treatment groups tested and symbol color indicates if the beta diversity was found to be statistically different (PERMANOVA test). P-values were adjusted by using the false discovery rate method for multiple comparisons. BMD treatment resulted in different cecal bacterial communities at every time point considered. Panel upper labels indicate days after start of BMD diets. ctrl, no antibiotic control; sub, subtherapeutic BMD dose; ther, therapeutic BMD dose.

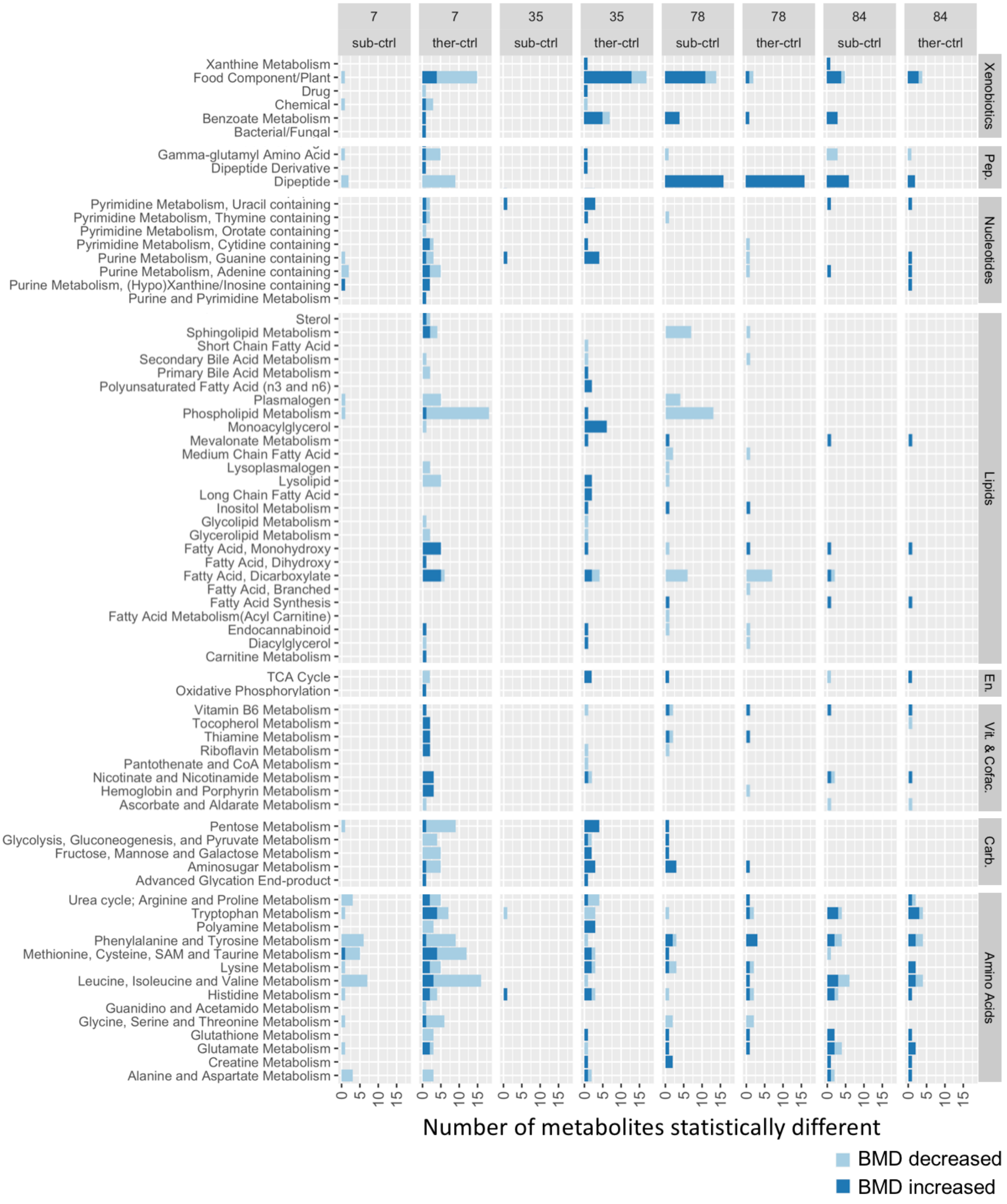


**Fig. S8:** Counts of cecal metabolites that were differentially (q < 0.1) present in the animals given subtherapeutic or therapeutic in feed BMD compared to the control animals. Metabolites are grouped by compound classes. Panel upper labels indicate days after start of BMD diets and treatment group comparison. ctrl, no antibiotic control; sub, subtherapeutic BMD dose; ther, therapeutic BMD dose.

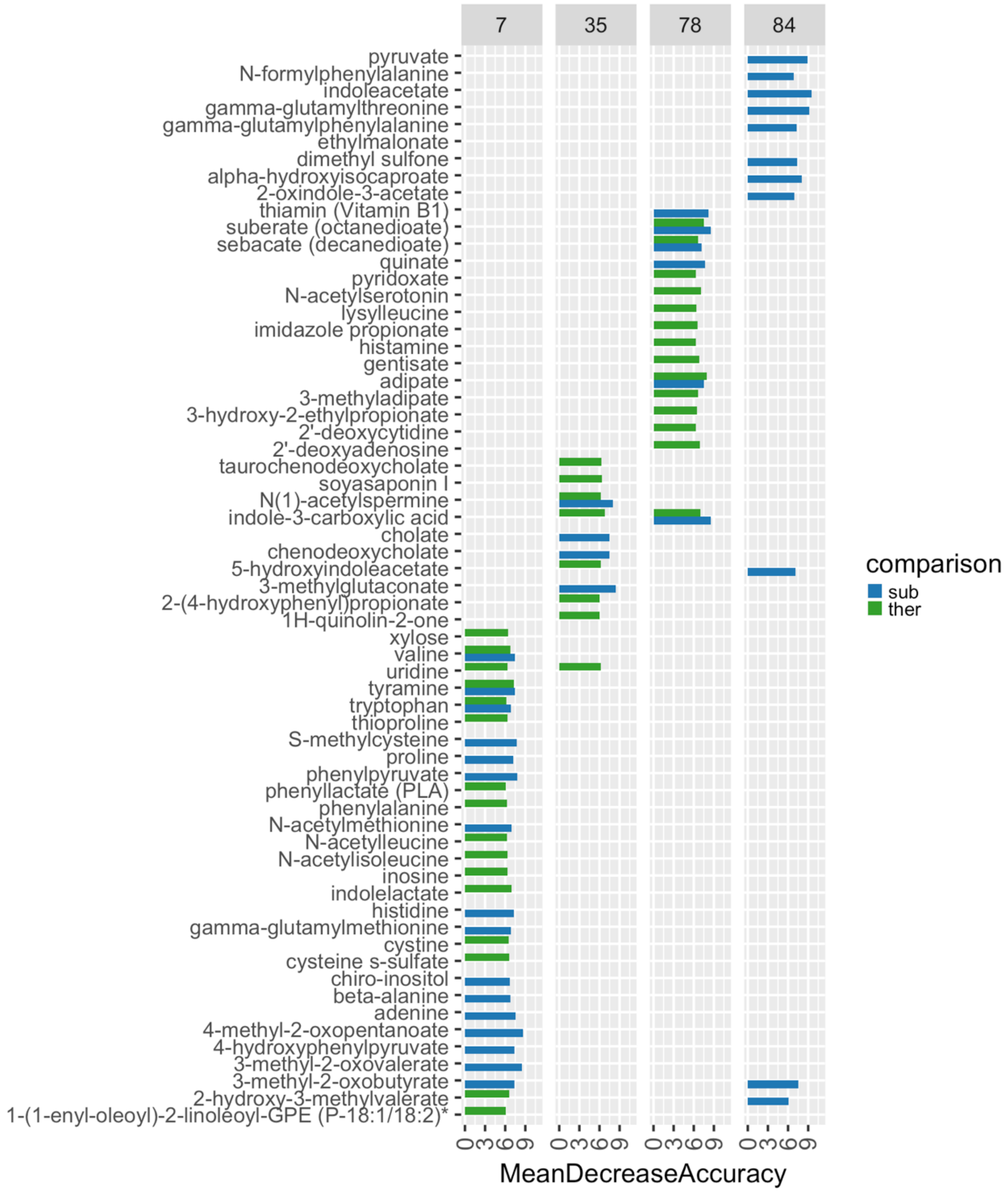


**Fig. S9:** Top metabolites in differentiating metabolomes from control birds from those given BMD according to random forest analysis. All metabolites with a mean decrease accuracy statistic greater than 6 are shown. A mean decrease accuracy of 6 was found to be a natural breakpoint in all treatment groups between high and low mean decrease accuracy metabolites. Panel upper labels indicate days after start of BMD diets. ctrl, no antibiotic control; sub, subtherapeutic BMD dose; ther, therapeutic BMD dose.

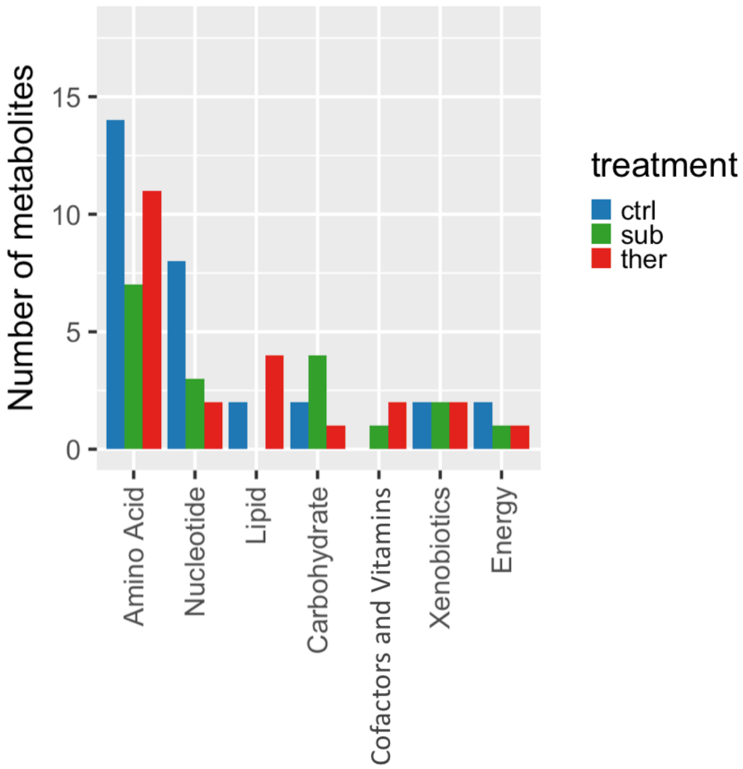


**Fig. S10:** Total numbers of metabolites whose concentration was well predicted by the microbial community composition by MIMOSA. Well-predicted metabolites are those for the community-based metabolite potential (CMP) scores were significantly correlated with measured metabolite abundance (using a Mantel test) with a false discovery rate (FDR) of ≤ 0.01. ctrl, no antibiotic control; sub, subtherapeutic BMD dose; ther, therapeutic BMD dose.

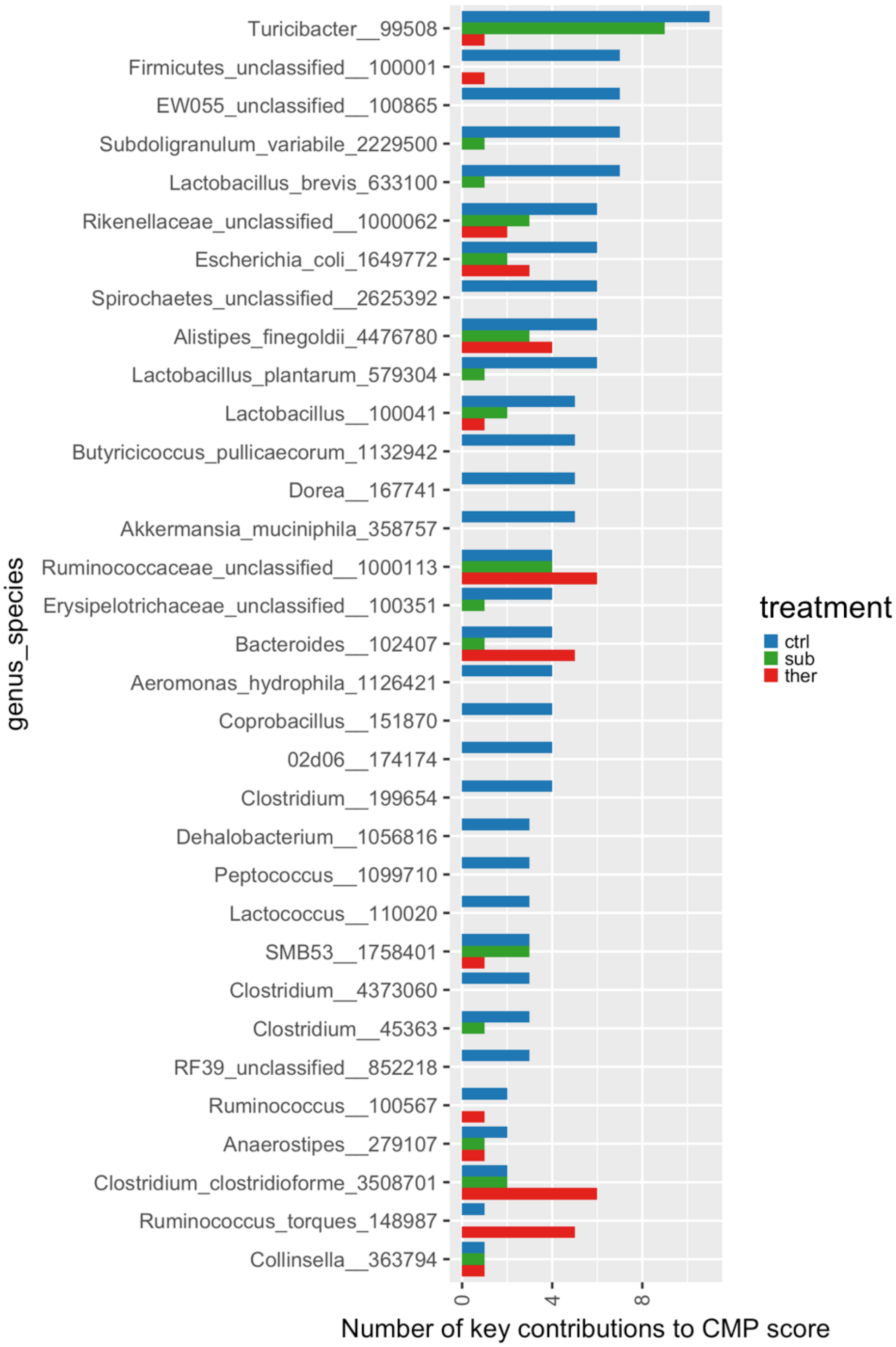


**Fig. S11:** The number of metabolites for which each species (best matched to OTU) that contributed to the calculation of CMP scores, as determined by MIMOSA analysis. ctrl, no antibiotic control; sub, subtherapeutic BMD dose; ther, therapeutic BMD dose.

**Supplementary Datasets:**

**Supplementary Data 1:** Results of metabolome profiling from all three treatment groups from days 7, 35, and 78. Data includes the original and scaled values. Results of the ANOVA testing are also showed in separate tabs.

**Supplementary Data 2:** Results of metabolome profiling from day 84 as well as the control group from day 78. Data includes the original and scaled values. Scaled data is then combined with that from days 7, 35, and 78. Results of the ANOVA testing are also showed in separate tabs.
